# Protective potential of miR-146a-5p and its underlying molecular mechanism in diverse cancers: a comprehensive meta-analysis and bioinformatics analysis

**DOI:** 10.1186/s12935-019-0886-y

**Published:** 2019-06-24

**Authors:** Mei-wei Li, Li Gao, Yi-wu Dang, Ping Li, Zu-yun Li, Gang Chen, Dian-zhong Luo

**Affiliations:** Department of Pathology, First Affiliated Hospital of Guangxi Medical University, 6 Shuangyong Road, Nanning, 530021 Guangxi Zhuang Autonomous Region P. R. China

**Keywords:** miR-146a-5p, Cancer, Prognosis, Meta-analysis, Molecular mechanism

## Abstract

**Background/aims:**

Studies have shown that miR-146a-5p was differentially expressed in diverse cancers, but the associations between miR-146a-5p expression and prognosis across multiple types of cancer as well its potential targets and downstream pathways have not been comprehensively analyzed. In this study, we performed the first meta-analysis of the prognostic value of miR-146a-5p expression in diverse malignancies and explored prospective targets of miR-146a-5p and related signaling pathways.

**Methods:**

A thorough search for articles related to miR-146a-5p was performed, and RNA-seq data from The Cancer Genome Atlas (TCGA) and microarray data from gene expression omnibus profiles were used to collect information about the prognostic value of miR-146a-5p. A comprehensive meta-analysis was conducted. Twelve platforms in miRWalk 2.0 were applied to predict targets of miR-146a-5p. TCGA RNA-seq data were used to validate the inverse relationships between miR-146a-5p and its likely targets. Subsequently, gene ontology and pathway analyses were conducted using Funrich version 3.1.3. Potential protein–protein interaction (PPI) networks were constructed. Potential target genes of miR-146a-5p in lung cancer were validated by RT-qPCR.

**Results:**

We included 10 articles in the meta-analysis. In a pooled analysis, the high miR-146a-5p expression group showed a better overall survival in solid cancers, particularly in reproductive system cancers and digestive system cancers. A total of 120 predicted target genes were included in a bioinformatics analysis. Five pathways involving phospholipase C (PLC) and aquaporins (AQPs) were the most significantly enriched Kyoto Encyclopedia of Genes and Genomes pathways. Moreover, the PPI network displayed the related signaling pathways and interactions among proteins. AQP1 and FYN were validated by RT-qPCR to be potential targets of miR-146a-5p in lung cancer.

**Conclusion:**

There is a close link between high miR-146a-5p expression and better overall survival in 21 types of solid cancer, especially in reproductive system and digestive system cancers. Furthermore, miR-146a-5p could inhibit diverse malignancies by modulating pathways linked to PLC or AQPs. In summary, miR-146a-5p is a potential prognostic biomarker and therapeutic target for various cancers.

## Background

Cancer is a serious threat to human health, with almost 1,735,350 newly diagnosed cancer cases and nearly 609,640 cancer-related deaths in the United States in 2018 [1, 2]. Gene sequencing technologies are frequently utilized to explore correlations between genomic changes and morbidity from various cancers [2–4]. Multiple factors regulate tumorigenesis and tumor development by altering DNA replication, transcription, and translation. The discovery of these factors, including microRNAs (miRNAs) and long non-coding RNAs, was considered a breakthrough for the early diagnosis and prevention of cancers. The identification of new molecular biomarkers and studies of their underlying mechanisms are valuable for the development of more effective treatment strategies.

miRNAs are single-stranded non-coding RNAs (18–22 nucleotides) with vital roles in the regulation of biological processes, including transcription, translation, cell cycle, and organismal development [5]. These molecules are linked to post-transcriptional regulation by interacting with corresponding messenger RNAs (mRNAs). Deregulated expression by miRNAs could increase the risk of metabolic diseases, such as diabetes and obesity, by disrupting signaling pathways [6]. Moreover, numerous studies have reported associations between altered miRNAs and cancers in different systems, suggesting that miRNAs are potential biomarkers for early diagnosis, treatment, and prognosis in cancers [7–14]. Furthermore, miRNAs are promising therapeutic candidates for metastatic cancer [5].

MiR-146a-5p, a member of the microRNA-146 family, has a crucial role in a series of cancer-related processes, including tumorigenesis, tumor progression, and outcomes. The expression of miR-146a-5p is increased in tissue samples from breast cancer and thyroid carcinoma [15, 16]. By contrast, levels of miR-146a-5p are decreased in gastric cancer, lung cancer, and prostate cancer [17–20]. Decreased miR-146a-5p levels are associated with biological activities in the latter cancers (gastric, lung, and prostate cancers), such as the growth, invasion, and migration of cancer cells [21]. It has been demonstrated that miR-146a-5p is a powerful suppressor in breast cancer, lung cancer, and prostate cancer [18–20]. Increasing studies have provided insight into the mechanism underlying the effects of abnormal miR-146a-5p on various cancers. A recent study has shown that down-regulated miR-146a-5p in gastric cancer is associated with poor prognosis and *WASF2* might be a target gene of miR-146a-5p [17]. However, more detailed analyses of the correlation between differentially expressed miR-146a-5p and prognosis for other solid tumors are needed. Therefore, we performed a comprehensive and thorough analysis of its prognostic significance by utilizing integrated data extracted from the literature, RNA-seq data from The Cancer Genome Atlas (TCGA) datasets, and the SurvMicro website. Additionally, to examine the mechanism underlying the effects of aberrant miR-146a-5p in solid cancers, a pathway analysis and protein interaction network analysis were conducted.

## Materials and methods

### Literature search strategy

A systematic searched for literature related to the prognostic value of miR-146a-5p in cancer was performed using the PubMed, EBSCO, CNKI, VIP, and WanFang databases. The most recent search was performed on July 16, 2017. The search terms for English-language databases included “miR146”, “miRNA146”, “microRNA146”, “microRNA146a”, “miR146a”, “miRNA146a”, “microRNA-146a-5p”, “miRNA-146a-5p”, and “miR-146a-5p” as well as “cancer”, “carcinoma”, “adenocarcinoma”, “sarcoma”, “tumor”, “neoplas*”, and “malignan*”, using “OR” to connect terms. Finally, “AND” was used to link the two classes of terms. For searches against Chinese databases, similar terms were input. Two authors performed the search independently to ensure the accuracy.

### Eligibility criteria

Only studies that satisfied the following criteria were included in the meta-analysis: (1) samples were obtained from human tissues or blood; (2) clearly described analysis of miR-146a-5p; (3) explored the prognostic value of miR-146a-5p expression levels in cancers; (4) provided sufficient information to extract hazard ratios (HRs) and 95% confidence intervals (CIs). The exclusion criteria were as follows: (1) unrelated to humans; (2) neither Chinese nor English, reviews, conference abstracts, case reports; (3) unable to extract HR and 95% CIs; (4) not satisfying the inclusion criteria.

### Data extraction

Data extraction was performed by two reviewers independently. The following information was extracted: the name of the first author, country, publication year, tumor type, number of cases, tumor stage, lymph node metastasis, time of follow-up, sample type, miR-146a level, cut-off values, HR and corresponding 95% CI. Comprehensive discussions were conducted to resolve any disagreements.

### Prognostic data for 21 human cancers downloaded from RNA-seq data

Expression levels of miR-146a-5p and corresponding prognostic data were obtained for 21 types of solid cancers from RNA-seq data. Expression values of less than 1 were removed. The median expression values were calculated using SPSS 22.0. Then, each cohort was separated into an experimental group (high expression level) and control group (low expression level). GraphPad Prism 7.0 was utilized to draw survival curves for 21 solid cancers. Cox regression was employed to calculate HRs.

### Statistical analysis

Stata12.0 and SPSS 22.0 were used to conduct statistical analyses. HRs and 95% CIs were extracted from the literature, RNA-seq data, and microarray profiles to obtain pooled results. HRs were directly collected if the they were reported in studies. HRs and 95% CIs were calculated based on the methods described by Tierney when the information provided was insufficient. Otherwise, Engauge Digitizer version 4.1 was used for studies that did not report concrete data. This software could determine HRs and 95% CIs based on Kaplan–Meier survival curves. The prognostic data downloaded from TCGA and SurvMicro were analyzed using SPSS 22.0 to estimate HRs and 95% CI. An HR greater than 1 indicated that patients with high miR-146a-5p expression were more likely to have a poor prognosis.

An integrated meta-analysis of relevant literature studies, RNA-seq data, and microarray profiles as well as separate meta-analysis for literature studies and RNA-seq data were performed. Heterogeneity was evaluated by the *I*^2^ statistic. Subgroup analysis stratified by human systems was used to identify the source of heterogeneity. Depending on the heterogeneity results, two distinct models were adopted, a random-effects model or fixed-effects model. A random-effects model was applied when there was obvious statistical heterogeneity; a fixed-effects model was applied in other cases. A significant difference was observed when the two-sided p-value was less than 0.05.

### Target prediction and validation for miR-146a-5p

Target genes for miR-146a-5p were predicted using MirWalk2.0. Twelve algorithms were used, including miRWalk, Microt4, miRanda, mirBridge, miRDB, miRMap, miRNAMap, Pictar2, PITA, RNA22, RNAhybrid, and TargetScan [22, 23]. According to the miRWalk2.0 algorithm, genes would be recognized as potential targets if there was a seed sequence that binds to miRNAs. The sequence of the genes could be complementary to that of miRNAs in the 3′-untranslated region, 5′-untranslated region, promoter, or coding sequence [22, 23]. To improve the accuracy of the prediction analysis, genes that overlapped in more than seven databases were chosen. Another criterion was applied to select the potential targets based on expression levels. A list of differentially expressed genes (DE genes) for 21 types of solid cancer was obtained from the TCGA and GTEx databases using GEPIA (|Log2FC| > 2, q-value < 0.05, limma methods) [24]. Because miR-146a-5p targets might show differential expression in diverse tumors, the intersection of predicted genes and DE genes in solid cancers contained the most likely potential targets of miR-146a-5p.

To further validate the relationships between miR-146a-5p and the potential targets, the correlations between their expression levels were assessed using R with miRNA sequence data [log2 (RPM + 1), miRNA mature strand expression] and RNA sequence data [log2 (TPM + 0.001), gene expression RNAseq] for the TCGA Pan-Cancer cohort with 7965 tumor samples and 639 non-tumor adjacent-tumor samples. To obtain reliable results, these data were first processed as follows: Relative expression = Expression in the cancer tissue group—mean expression in the non-cancer tissue group. Ultimately, the genes were selected as candidate targets when their expression levels were inversely associated with levels of miR-146a-5p (correlation coefficient < 0, p < 0.05), and these genes were used to explore the mechanism underlying the effects of miR-146a-5p in diverse cancers.

### Bioinformatics analysis

The molecular mechanism underlying the effect of aberrant miR-146a-5p levels in solid cancers was evaluated using Funrich version 3.1.3, with the assistance of gene ontology (GO) and pathway analyses. By observing the p-value for each term, significant pathways were identified and the molecular functions, biological processes, and cellular components involved in the effects of miR-146a-5p were explored. The search tool for the retrieval of interacting genes/proteins (STRING) database (http://string-db.org/) was used for the construction of the protein–protein interaction (PPI) network, which displayed potential signaling pathways and connections among proteins in a dimensional way. The potential binding sites of miR-146a-5p and candidate targets were determined using miRWalk2.0. Additionally, scatter diagrams were constructed to visualize correlations between the expression levels of miR-146a-5p and candidate targets in diverse malignant tumors, utilizing miRNA and RNA sequence data.

### Validation of potential targets of miR-146a-5p through RT-qPCR

Previously, we found that miR-146a-5p showed a decreased expression in lung cancer tissues compared to normal lung tissues [25]. Then we collected 55 lung cancer tissues (32 lung adenocarcinoma and 23 lung squamous cell carcinoma) and their matched non-tumor lung tissues to further test the expression of miR-146a-5p potential targets (AQP1 and FYN). The mean age of the patients was 56.9 years old. The Ethical Committees of First Affiliated Hospital, Guangxi Medical University, China have approved the study protocol. And all of the patients have signed the informed consent. According to the methods described in our previous studies [26–32], we isolated total RNA and performed relative quantification analysis. RT–qPCR was executed using the 7900HT PCR system of USA (Applied Biosystems; Thermo Fisher Scientific, Inc., Waltham, MA). The internal control in the analysis was GAPDH, with the forward primer (5′-TGCACCACCAACTGCTTA-3′) and the reverse primer (5′-GGATGCAGGGATGATGTTC-3′). The thermocycling steps included hot start at 95 °C for 10 min, 95 °C for 10 s, 60 °C for 5 s and annealing at 72 °C for 5 s, totally 40 PCR cycles. And the 2^−∆Cq^ method was utilized to calculate expression of candidate miR-146a-5p targets.

## Results

### Characteristics of the studies included in the meta-analysis

**Fig. 1:**
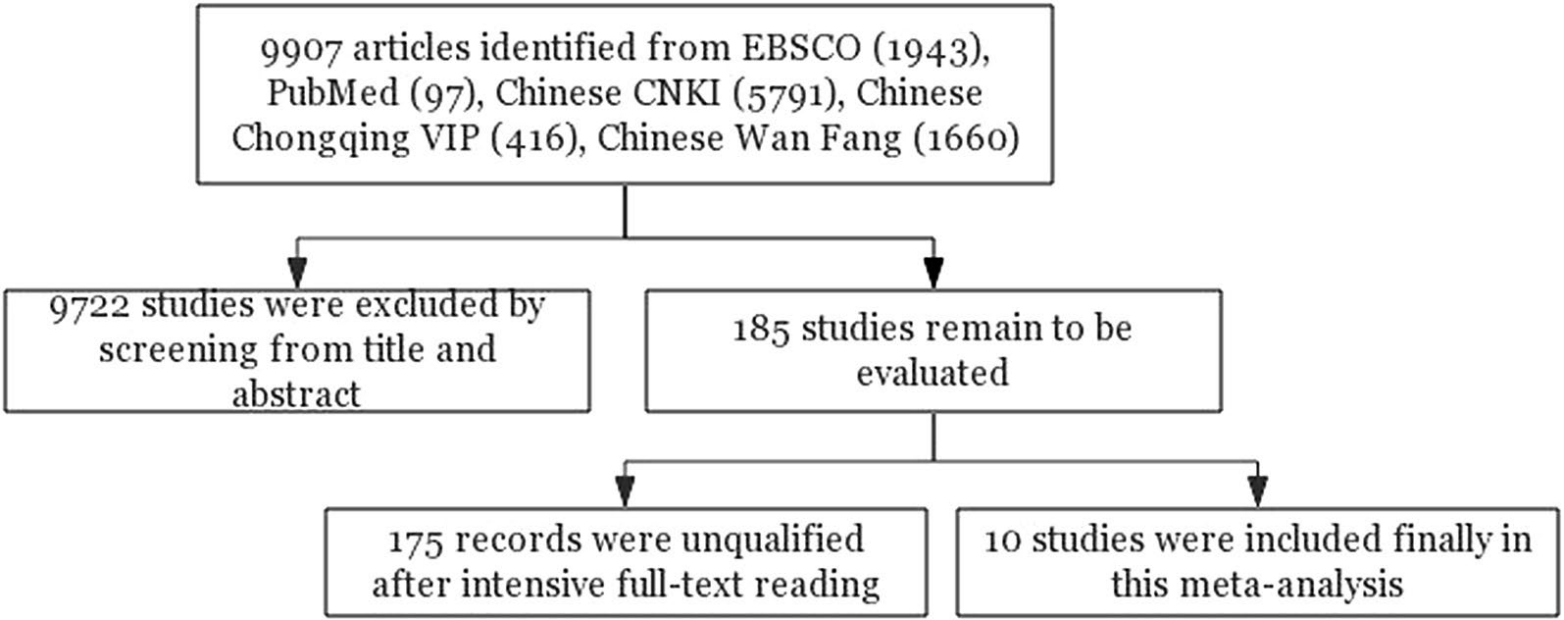
Flow diagram of the process for searching for articles

A total of 9907 references were collected in the initial search, of which 9722 were removed based on titles and abstracts. After full-text screening, we included ten studies published between 2010 and 2017 in the meta-analysis, with 783 patients in total (Fig. 1) [25, 33–41]. Tumor tissues were mostly utilized to detect miR-146a-5p expression, and bone marrow samples were employed in one of the included studies. The necessary information about the ten studies is outlined in Table 1.

**Table 1:** Main characteristics of the articles included in the meta-analysis

Study	Year	Country	Tumour type	N	Detected sample	Stage I/II/III/IV	Lymph node −/+	Follow-up months	miR-146a assay	Cut-off	HR (95% CI)
Hess	2017	Germany	HNSCC	149	Tissue	0/0/0/149	NR	Median 61	qRT-PCR	Median	0.450 (0.230–0.830)
LUO	2017	China	GC	93	Tissue	0/0/42/51	17/76	NR	qRT-PCR	2.0	0.129 (0.028–0.601)
Zavala	2016	Chile	TNBC	39	Tissue	NR	20/19	NR	qRT-PCR	Median	0.130 (0.026–0.650)
Li	2014	China	NSCLC	43	Tissue	NR	NR	NR	MISH & qRT-PCR	Median	2.930 (1.440–5.960)
Chen	2013	China	NSCLC	101	Tissue	(I/II:28) (III/IV:73)	35/66	NR	qRT-PCR	Mean	0.420 (0.180–0.950)
Zhong	2012	China	DLBCL	90	Tissue	9/31/24/26	NR	Mean 26.3	qRT-PCR	Optimal	1.119 (0.977–1.282)
Hou	2012	China	GC	43	Tissue	8/11/18/6	14/29	NR	qRT-PCR	Median	0.280 (0.100–0.750)
Paik	2011	Korea	NK/T	50	Tissue	26/11/0/13	43/7	NR	qRT-PCR	Optimal	0.076 (0.019–0.300)
Kogo	2011	Japan	GC	90	Tissue	27/18/23/22	30/60	Mean 36	qRT-PCR	Median	0.650 (0.440–0.940)
Wang	2010	China	ALL	32	Bone marrow	NR	NR	NR	qRT-PCR	Median	1.690 (1.040–2.760)
Wang	2010	China	AML	53	Bone marrow	NR	NR	NR	qRT-PCR	Median	1.560 (1.120–2.170)

HNSCC: head and neck squamous cell carcinoma; GC: gastric cancer; TNBC: triple negative breast cancer; NSCLC: non-small cell lung cancer; DLBCL: diffuse large B-cell lymphoma; NK/T: NK/T cell lymphoma; ALL: acute lymphoblastic leukemia; AML: acute myeloid leukemia; NR: not reported; median: middle value; mean: average value; qRT-PCR: quantitative real-time polymerase chain reaction; MISH: miRNA in situ hybridization; optimal: cut-off point associated with a minimum p-value; HR: hazard ratio

### Survival curves

**Fig. 2:**
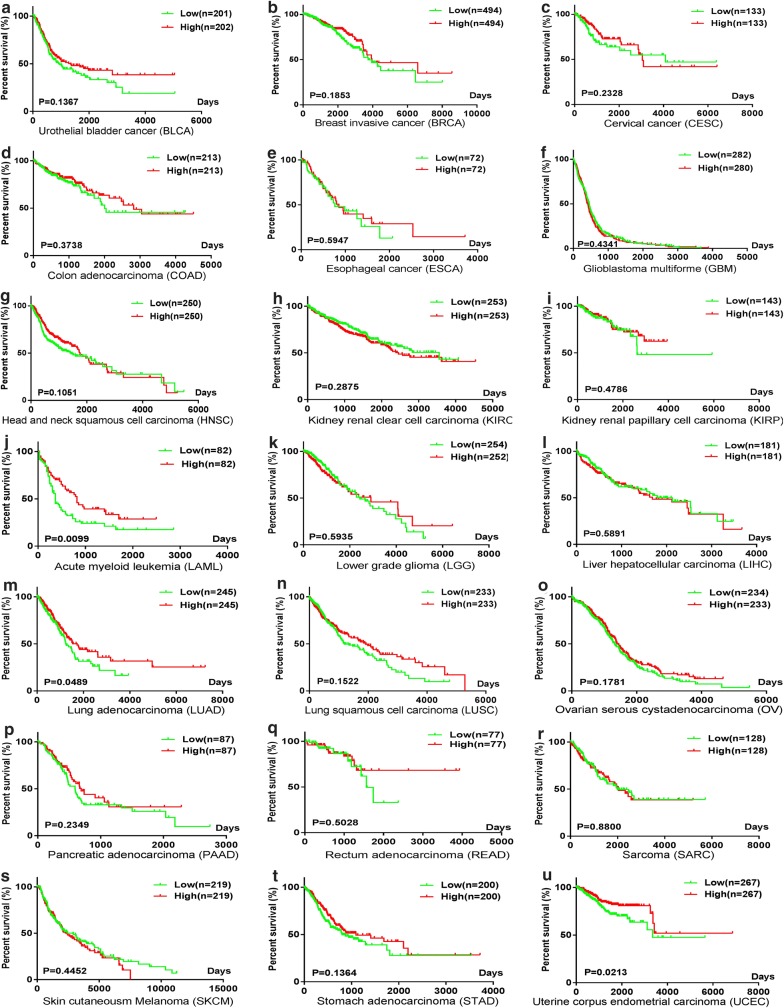
Kaplan–Meier survival curves for miR-146a-5p in diverse cancers using RNA-seq data

The expression and survival data for miR-146a-5p in 21 types of solid cancers were extracted from RNA-seq data, including data for 8519 patients. HRs were estimated using SPSS 22.0 and the results are summarized in Table 2. Kaplan–Meier survival curves were utilized to compare survival between low and high miR-146a-5p expression groups using GraphPad Prism 7.0 (Fig. 2).

**Table 2:** Characteristics of eligible studies from RNA-seq data and microarray profiles

Study	Cancer type	Group	Number	HR
TCGA BLCA	Urothelial bladder cancer	Urinary system	404	0.800 (0.595–1.074)
TCGA BRCA	Breast invasive cancer	Endocrine system	988	0.793 (0.562–1.119)
TCGA CESC	Cervical cancer	Reproductive system	266	0.736 (0.444–1.220)
TCGA COAD	Colon adenocarcinoma	Digestive system	426	0.834 (0.559–1.245)
TCGA ESCA	Esophageal cancer	Digestive system	144	0.869 (0.517–1.460)
TCGA GBM	Glioblastoma multiforme	Nervous system	562	1.075 (0.896–1.290)
TCGA HNSC	Head and neck squamous cell carcinoma	Other	500	0.800 (0.611–1.048)
TCGA KIRC	Kidney renal clear cell carcinoma	Urinary system	506	1.177 (0.871–1.592)
TCGA KIRP	Kidney renal papillary cell carcinoma	Urinary system	286	0.805 (0.442–1.468)
TCGA LAML	Acute myeloid leukemia	Other	164	0.596 (0.400–0.890)
TCGA LGG	Lower grade glioma	Nervous system	506	1.101 (0.773–1.569)
TCGA LIHC	Liver hepatocellular carcinoma	Digestive system	262	1.102 (0.775–1.567)
TCGA LUAD	Lung adenocarcinoma	Respiratory system	262	0.743 (0.552–1.000)
TCGA LUSC	Lung squamous cell carcinoma	Respiratory system	466	0.815 (0.615–1.079)
TCGA OV	Ovarian serous cystadenocarcinoma	Reproductive system	470	0.855 (0.680–1.075)
TCGA PAAD	Pancreatic adenocarcinoma	Digestive system	174	0.776 (0.509–1.181)
TCGA READ	Rectum adenocarcinoma	Digestive system	154	0.765 (0.348–1.680)
TCGA SARC	Sarcoma	Other	258	1.031 (0.691–1.539)
TCGA SKCM	Skin Cutaneous Melanoma	Other	438	1.113 (0.845–1.467)
TCGA STAD	Stomach adenocarcinoma	Digestive system	400	0.790 (0.579–1.078)
TCGA UCEC	Uterine corpus endometrial carcinoma	Reproductive system	534	0.608 (0.396–0.932)
GSE10694	Liver hepatocellular carcinoma	Digestive system	156	0.900 (0.620–1.310)
GSE13937	Lung cancer	Respiratory system	152	1.360 (0.830–2.220)
GSE16025	Lung cancer	Respiratory system	61	0.620 (0.310–1.220)
GSE21849	Acute myeloid leukemia	Other	37	0.340 (0.070–1.750)
GSE27290	Ovarian serous cystadenocarcinoma	Reproductive system	62	0.870 (0.460–1.640)
GSE27705	Lung cancer	Respiratory system	20	0.250 (0.070–0.960)
GSE31384	Liver hepatocellular carcinoma	Digestive system	166	1.410 (0.890–2.250)
GSE36682	Nasopharyngeal carcinoma	Respiratory system	62	1.030 (0.450–2.380)
GSE37405-GPL13703	Breast invasive cancer	Endocrine system	60	1.560 (0.740–3.330)
GSE37405-GPL14149	Breast invasive cancer	Endocrine system	40	0.800 (0.300–2.130)

### Pooled meta-analysis results

**Fig. 3:**
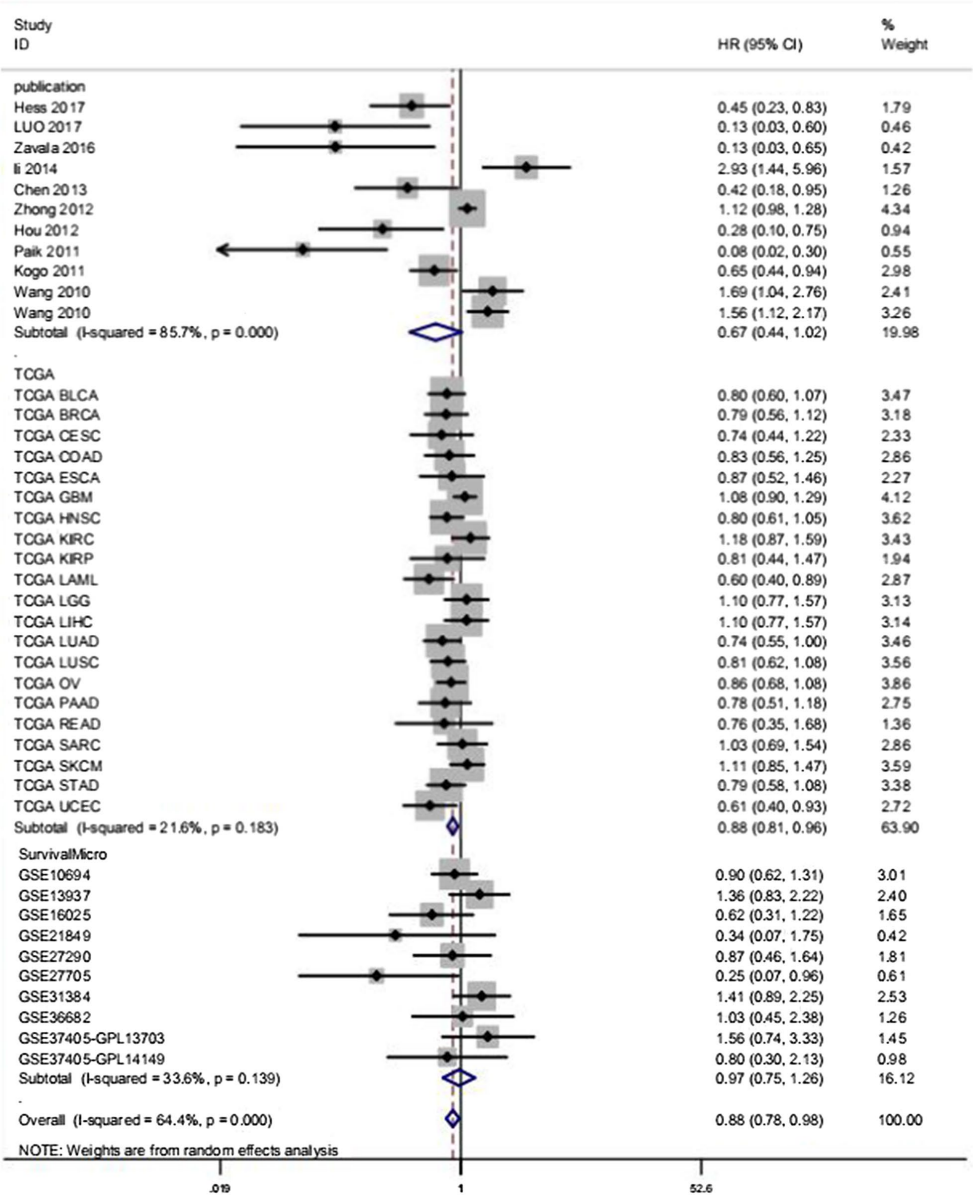
Subgroup analysis of total records to determine the prognostic value of miR-146a-5p in solid cancers. Generally, miR-146a-5p was expressed at low levels in diverse solid cancers and was related to a worse prognosis for patients with cancer (HR 0.875, 95% CI 0.784–0.976). Due to obvious heterogeneity (*I*^2^ = 64.4%), a random-effects model was used, as indicated in the note shown at the bottom

**Fig. 4:**
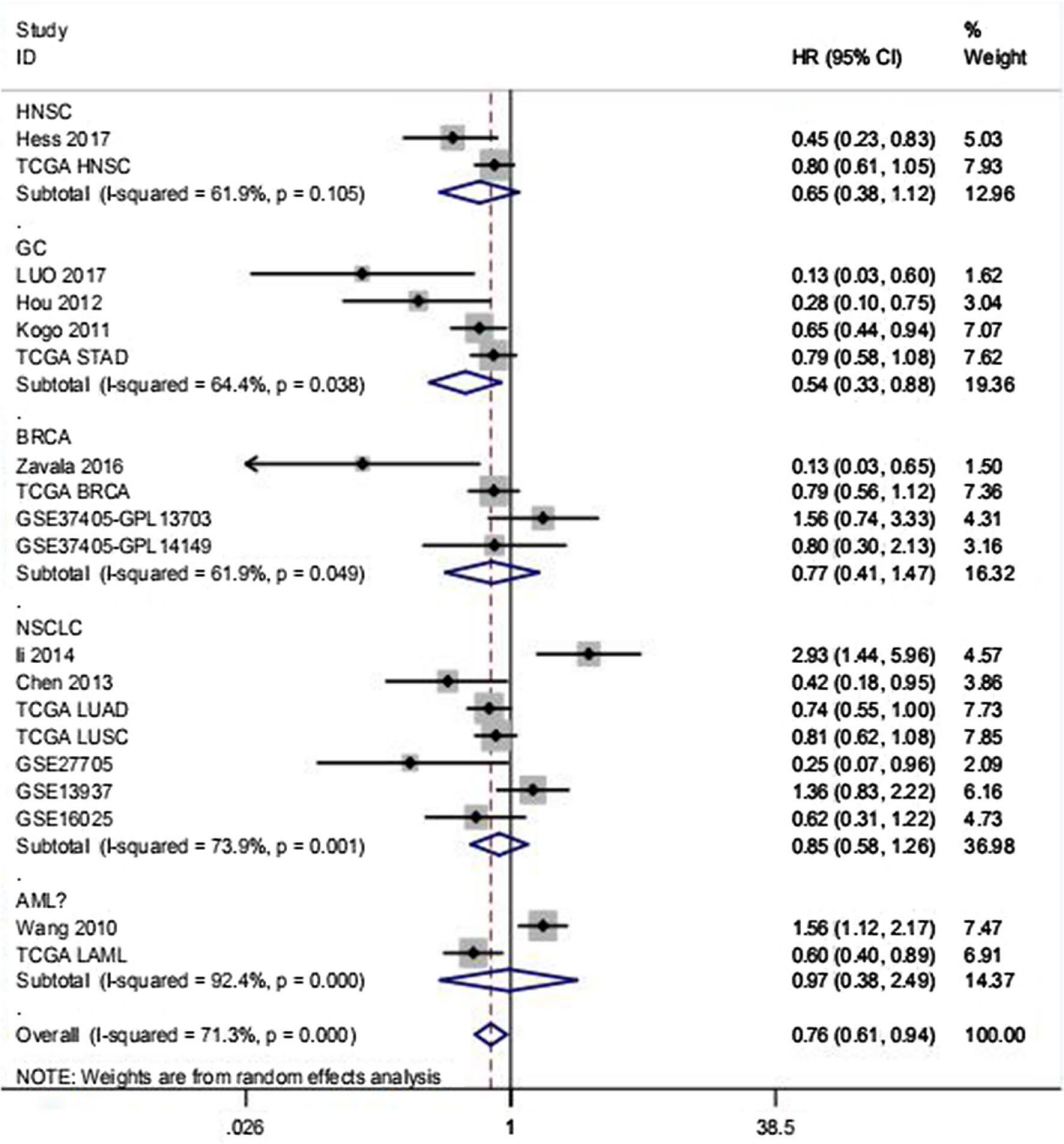
Subgroup analysis by tumor type to show the prognostic value of miR-146a-5p in diverse tumors. In the subgroup analysis, decreased miR-146a-5p expression in gastric cancer (GC) was associated with shorter overall survival (HR 0.535, 95% CI 0.327–0.877). The random-effects model was applied as noted above (*I*^2^ = 64.40%)

A comprehensive meta-analysis of relevant studies, RNA-seq data, and microarray expression profiles was conducted to analyze the prognostic value of miR-146a-5p in solid cancers. The results obtained using the total data (HR 0.875, 95% CI 0.784–0.976, *I*^2^ = 64.40%) suggested that the high miR-146a-5p group had a better overall survival than that of the low expression group (Fig. 3). Since significant heterogeneity was observed, we used the random-effects model. We obtained similar pooled results in subgroup analyses stratified by human systems (Table 3). Moreover, we found that increased miR-146a-5p levels were closely related to a better overall survival in reproductive system cancers (HR 0.791, 95% CI 0.661–0.947, *I*^2^ = 0.00%) and digestive system cancers (HR 0.844, 95% CI 0.738–0.965, *I*^2^ = 49.00%) (Table 3). Additionally, an association between high expression miR-146a-5p and a better prognosis was observed in gastric cancer (HR 0.535, 95% CI 0.327–0.877, *I*^2^ = 64.40%, random-effects model) (Table 3, Fig. 4).

**Table 3:** Summary of meta-analysis results for overall survival

Analysis	No. of studies	HR (95% CI)		Heterogeneity	
		Fix	Random	I^2 (^%)	p
Overall pooled result	42	0.940 (0.888–0.995)	0.875 (0.784–0.976)	64.40	0.000
*Data resource*					
The included studies	11	1.050 (0.941–1.172)	0.667 (0.438–1.016)	85.70	0.000
TCGA data	21	0.89 (0.830–0.956)	0.880 (0.810–0.956)	21.60	0.183
SurvMicro GSE	10	1.005 (0.825–1.224)	0.971 (0.747–1.261)	33.60	0.139
*Human system*					
Urinary system	3	0.947 (0.776–1.156)	0.938 (0.708–1.244)	43.40	0.171
Endocrine System	4	0.828 (0.618–1.111)	0.774 (0.406–1.474)	61.90	0.049
Reproductive system	4	0.791 (0.661–0.947)	0.791 (0.661–0.947)	0.00	0.560
Digestive system	11	0.844 (0.738–0.965)	0.821 (0.672–1.003)	49.00	0.033
Nervous system	2	1.08 (0.919–1.270)	1.080 (0.919–1.270)	0.00	0.906
Respiratory system	8	0.855 (0.723–1.011)	0.872 (0.611–1.244)	69.80	0.002
Other	10	1.036 (0.942–1.140)	0.895 (0.688–1.164)	79.60	0.000
*Tumour type*					
HNSC	2	0.734 (0.572–0.941)	0.648 (0.376–1.116)	61.90	0.105
GC	4	0.668 (0.530–0.841)	0.535 (0.327–0.877)	64.40	0.038
BRCA	4	0.828 (0.618–1.111)	0.774 (0.406–1.474)	61.90	0.049
NSCLC	7	0.848 (0.715–1.006)	0.853 (0.578–1.260)	73.90	0.001
AML	2	1.055 (0.818–1.362)	0.971 (0.378–2.492)	92.40	0.000
Overall pooled result		0.816 (0.737–0.904)	0.762 (0.614–0.945)	71.30	0.000

The pooled results for the 10 articles identified in the literature search showed no statistical significance, with obvious heterogeneity (HR 0.667, 95% CI 0.438–1.016, *I*^2^ = 85.70%) (Table 3). Thus, we used the random-effects model. However, a subgroup analysis by human system indicated that high expression of miR-146a-5p was associated with better prognosis in digestive system cancers (HR 0.355, 95% CI 0.143–0.881, *I*^2^ = 66.1%, random-effects model).

**Fig. 5:**
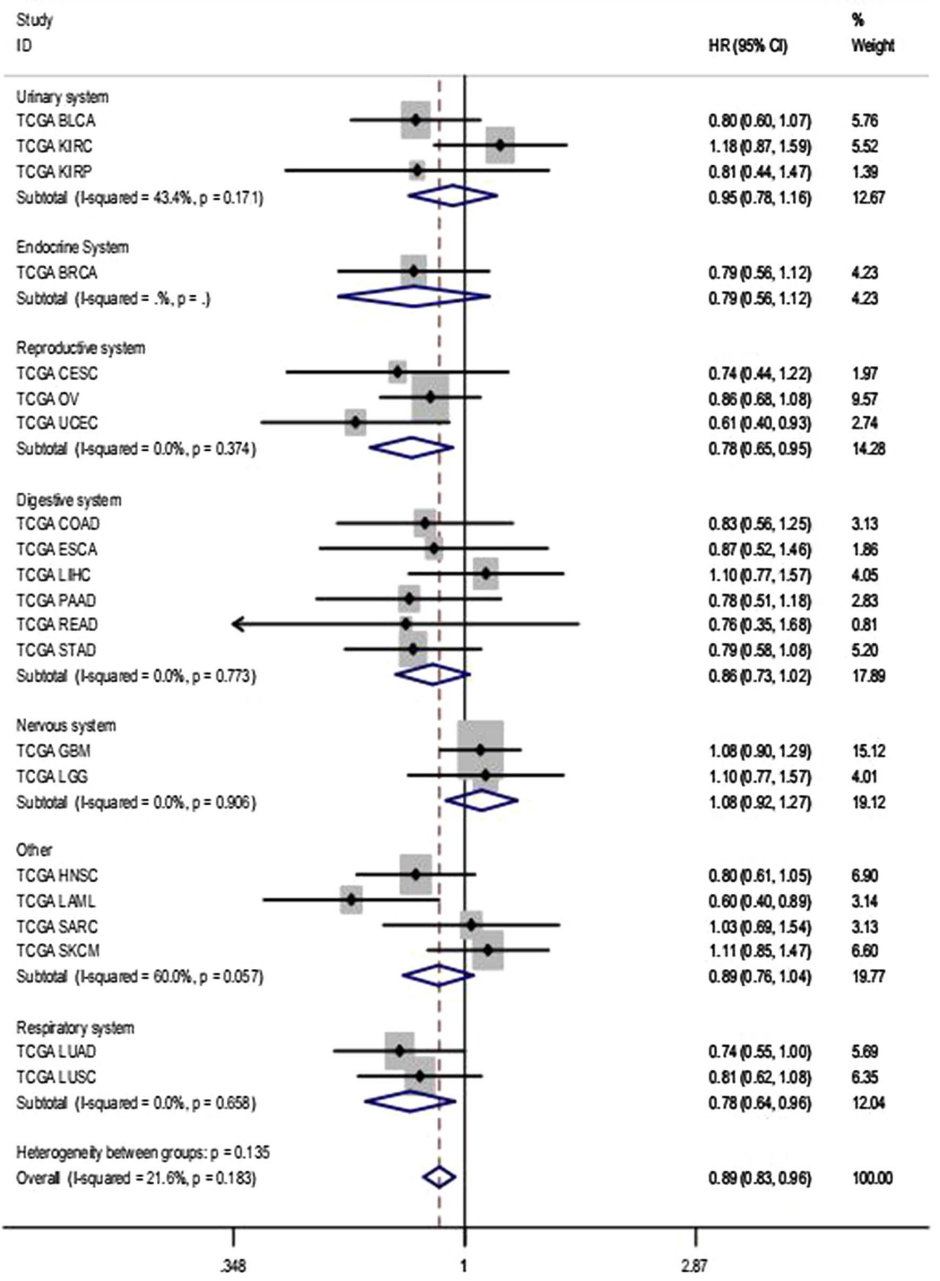
Subgroup analysis of TCGA data by human system. In combination with Table 3, lower miR-146a-5p expression was associated with poorer prognosis for patients with reproductive system cancers (HR 0.791, 95% CI 0.661–0.947, *I*^2^ = 0.00%) and digestive system cancers (HR 0.844, 95% CI 0.738–0.965, *I*^2^ = 49.00%). The final heterogeneity could be considered slight (*I*^2^ = 21.6%); Thus, a fixed-effect model was used

The aggregated results for RNA-seq data indicated that high miR-146a-5p expression was associated with a better prognosis in solid cancers without considerable heterogeneity (HR 0.880, 95% CI 0.810–0.956, *I*^2^ = 21.60%) (Table 3, Fig. 5). The association between high miR-146a-5p expression and improved survival was found in reproductive system cancers (HR 0.784, 95% CI 0.650–0.946, *I*^2^ = 0.00%) and respiratory system cancers (HR 0.780, 95% CI 0.636–0.957, *I*^2^ = 0.00%) (Fig. 5). There was no obvious link between miR-146a-5p and prognosis in other cancer types or systems.

### Target genes of miR-146a-5p

**Fig. 6:**
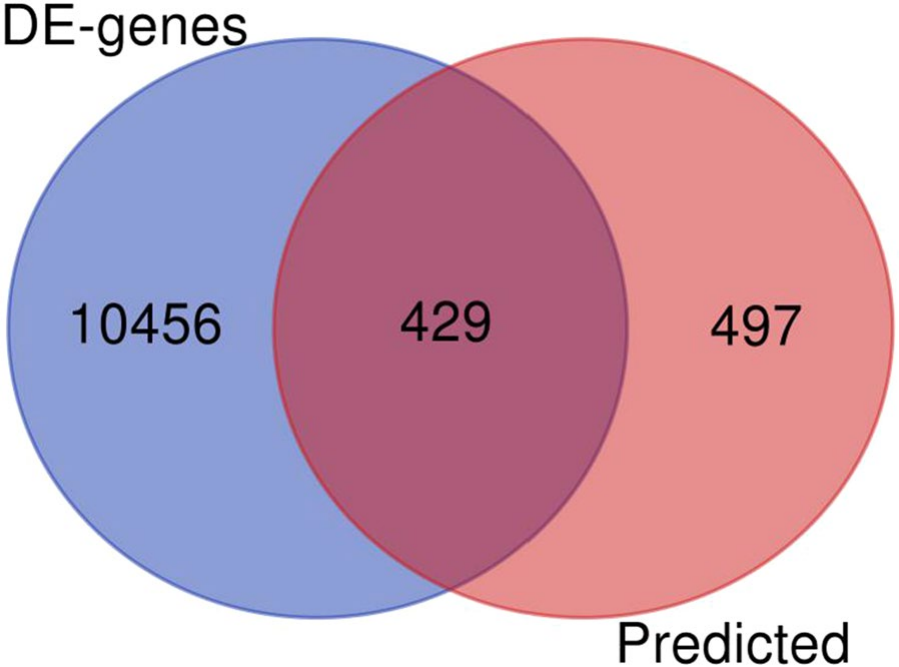
The 429 potential targets of miR-146a-5p. The genes were selected as potential targets based on sequence complementation and their expression levels in solid cancers

**Fig. 7:**
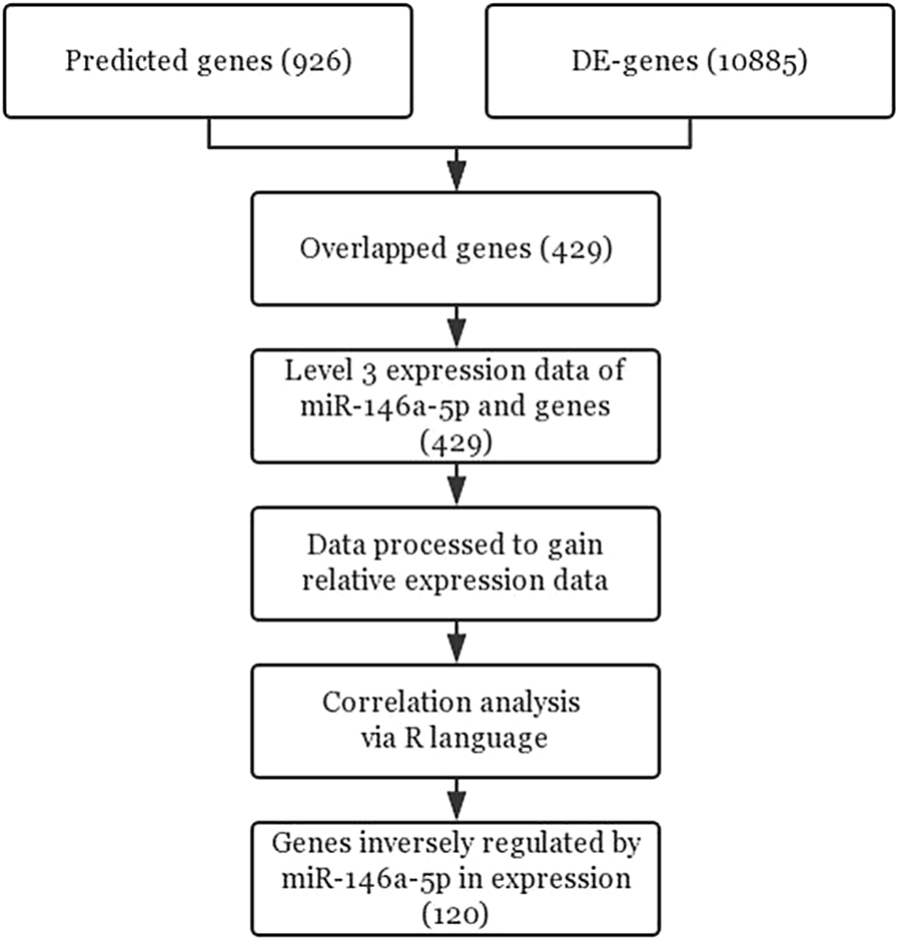
Flow chart of miR-146a-5p target prediction and validation. By utilizing twelve prediction platforms, DE genes from the GEPIA website and miRNA and RNA sequencing data for the TCGA Pan-Cancer cohort were used to identify 120 likely targets of miR-146a-5p

A total of 14,280 genes were identified as candidate targets using 12 prediction platforms, and only those targets identified by more than seven platforms were included in subsequent analyses. A total of 926 target genes were obtained, of which 429 were abnormally expressed in solid cancers (Fig. 6). Finally, expression levels of 120 genes were confirmed to be inversely correlated with the level of miR-146a-5p and thus these genes were identified as potential targets for subsequent analyses (Fig. 7).

### Bioinformatics analysis

**Fig. 8:**
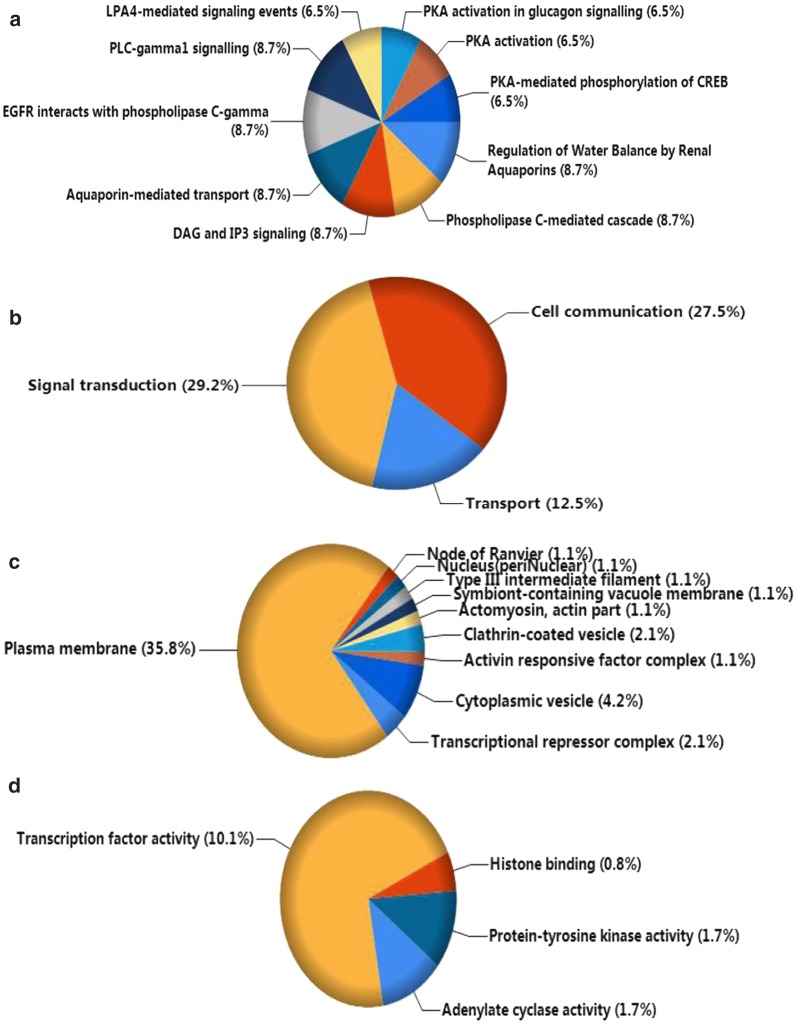
Visualization of the most highly enriched pathways and GO processes for miR-146a-5p targets. As described in Table 4, 120 potential targets were evaluated. miR-146a-5p was potentially involved in the regulation of following activities in various cancers: **a** KEGG pathways: PLC-mediated cascade, DAG and IP3 signaling, AQP-mediated transport, EGFR interactions with PLC-γ, and PLC-γ1 signaling; **b** biological processes: transport, signal transduction, cell communication; **c** cellular components: plasma membrane; **d** molecular function: transcription factor activity and protein-tyrosine kinase activity

**Fig. 9:**
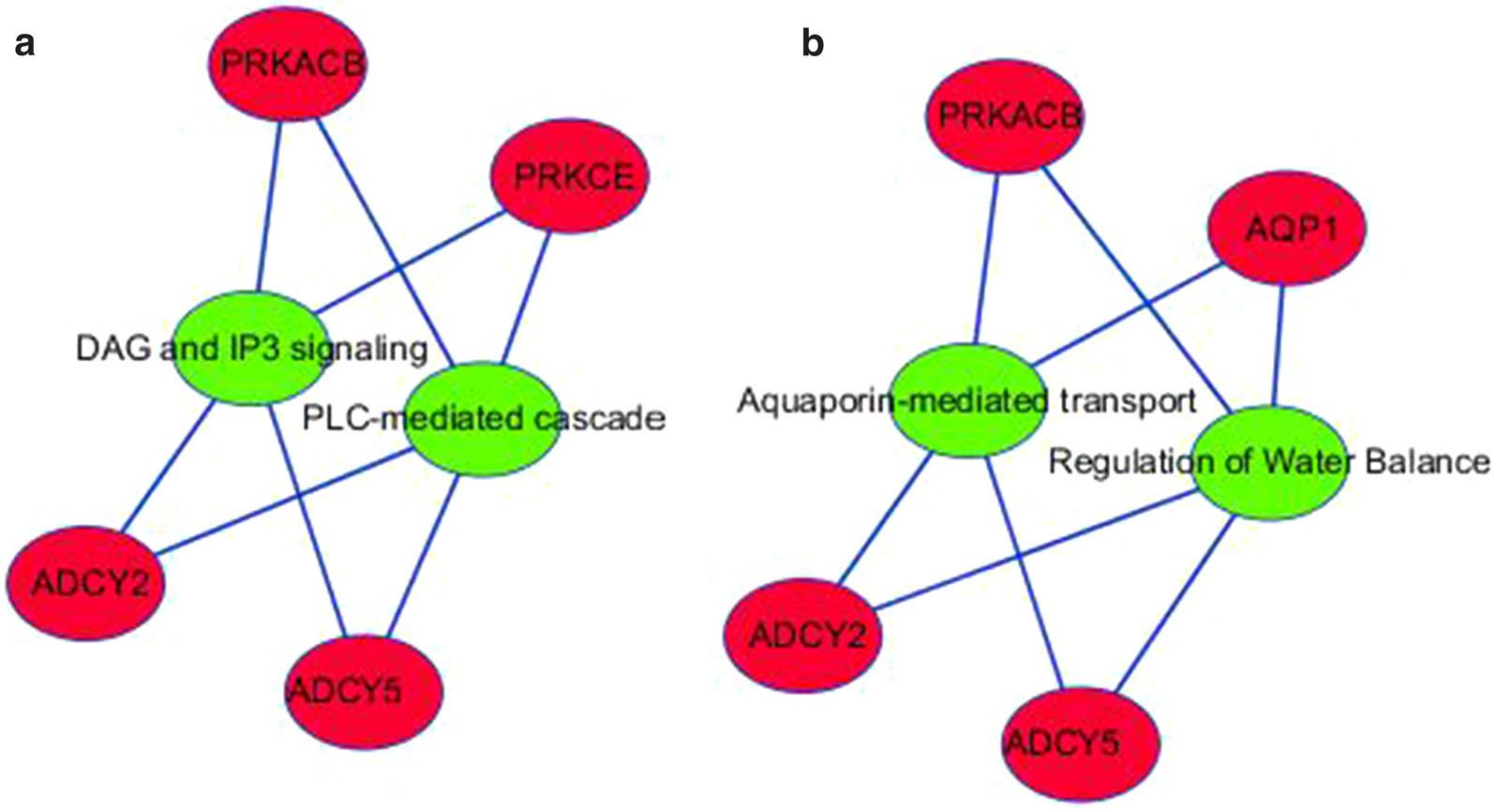
Connections among potential targets of miR-146a-5p in crucial pathways. **a** Pathways of PLC (green nodes); **b** pathways of AQPs (green nodes). *PRKACB*, *PRKCE*, *ADCY5*, *ADCY2*, and *AQP1* are five potentially vital genes (red nodes) in the above pathways

**Fig. 10:**
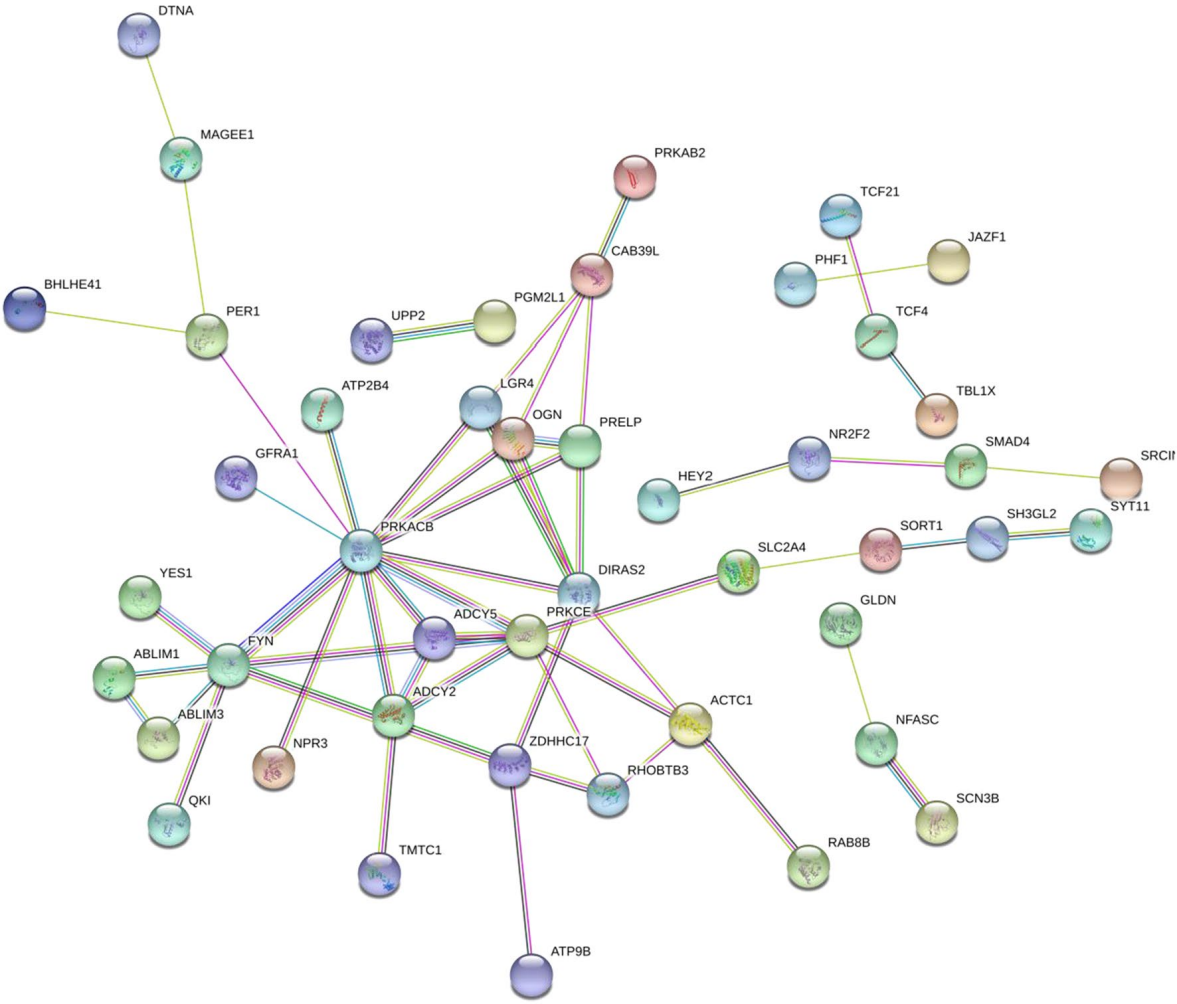
PPI network of miR-146a-5p potential target genes drawn using STRING. The most frequent genes were identified as hub genes, including *PRKACB*, *FYN*, and *PRKCE*

**Fig. 11:**
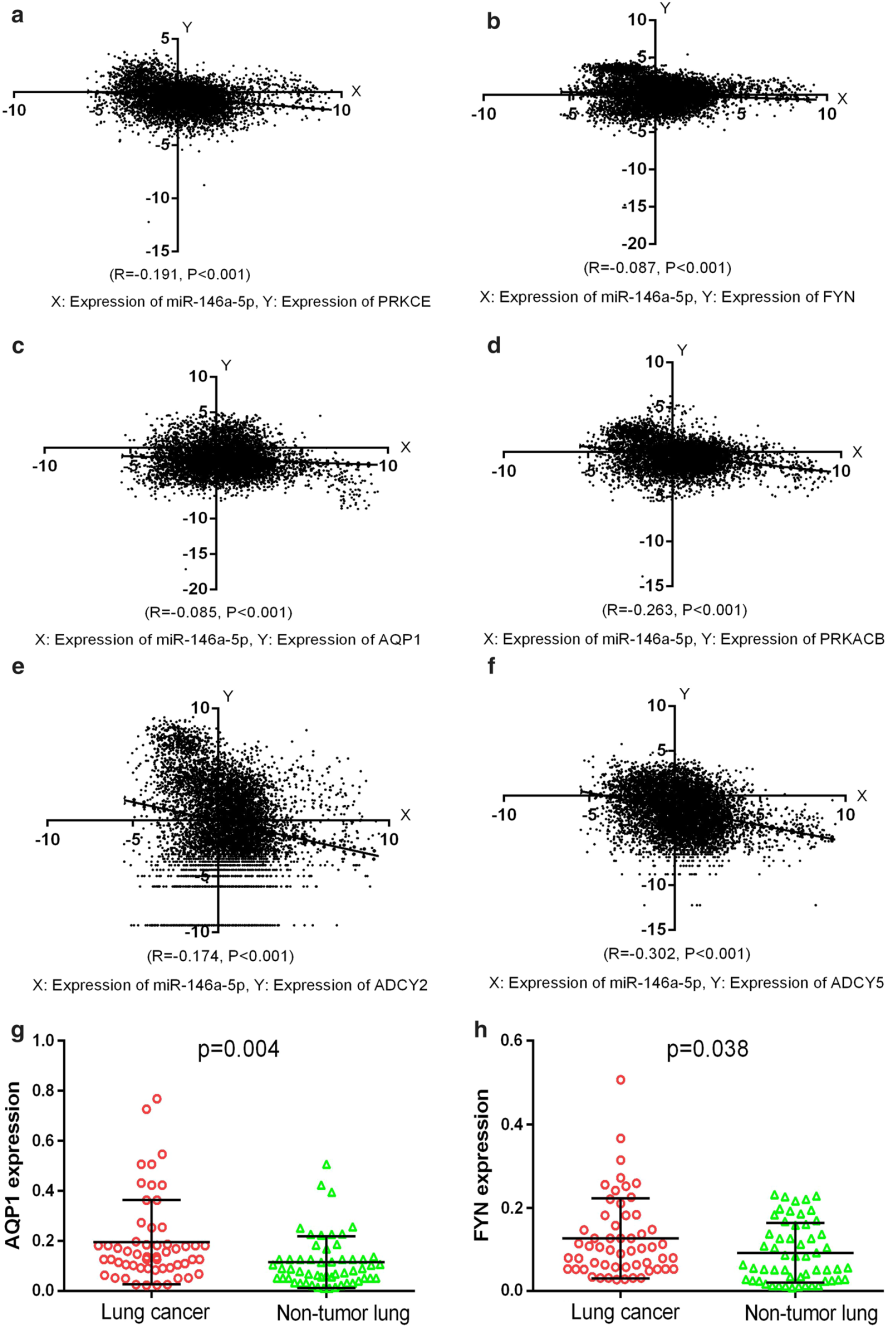
Correlations between expression levels of candidate targets and miR-146a-5p in pan-cancer. Expression data for miR-146a-5p and the six selected targets were obtained from the TCGA Pan-Cancer cohort, as above described. Expression levels of all candidate targets were inversely associated with miR-146a-5p expression. GraphPad Prism 6.0 was employed to generate scatter plots. **a** PRKCE; **b** FYN; **c** AQP1; **d** PRKACB; **e** ADCY2; **f** ADCY5. **g** AQP1 expression in lung cancer. **h** FYN expression in lung cancer

Funrich version 3.1.3 was utilized to perform GO and Kyoto Encyclopedia of Genes and Genomes (KEGG) pathway enrichment analyses using the 120 potential targets. In total, 126 biological functions were obtained in the GO analysis, of which 25 were significant (p < 0.05). Several processes might function as essential activities regulated by miR-146a-5p in various malignancies, including Transport, Signal transduction, and Cell communication in the biological processes category; Transcriptional repressor complex and Plasma membrane in the cellular components category; and adenylate cyclase activity, transcription factor activity, histone binding, and protein-tyrosine kinase activity in the molecular functions category (Fig. 8). A total of 291 critical pathways were identified by KEGG, and pathways involving phospholipase C (PLC) and aquaporins (AQPs) were the most statistically significant (p < 0.001), as shown in Table 4 and Fig. 8. In particular, *PRKACB*, *PRKCE*, *ADCY5*, *ADCY2,* and *AQP1* were identified as five key genes in the above pathways (Fig. 9). A protein network was constructed using STRING with 120 predicted targets, as shown in Fig. 10. The genes that frequently interacted with other genes were recognized as hub genes of miR-146a-5p, including *PRKACB*, *FYN*, and *PRKCE*. Overall, *PRKACB*, *PRKCE*, *ADCY5*, *ADCY2*, *AQP1,* and *FYN* were recognized as the most likely targets of miR-146a-5p. The predicted binding sites for the six genes and miR-146a-5p are displayed in Table 5. There were significant correlations between levels of the six likely targets and miR-146a-5p in solid cancers, as shown in Fig. 11.

**Table 4:** Most highly enriched pathways and processes for miR-146a-5p sorted by p-values

Terms	Count	Percentage (%)	p value
*KEGG pathways*			
Regulation of water balance by renal aquaporins	4	8.696	< 0.001
Phospholipase C-mediated cascade	4	8.696	< 0.001
DAG and IP3 signaling	4	8.696	< 0.001
Aquaporin-mediated transport	4	8.696	< 0.001
EGFR interacts with phospholipase C-gamma	4	8.696	< 0.001
PLC-gamma1 signalling	4	8.696	< 0.001
LPA4-mediated signaling events	3	6.522	< 0.001
PKA activation in glucagon signalling	3	6.522	< 0.001
PKA activation	3	6.522	< 0.001
PKA-mediated phosphorylation of CREB	3	6.522	< 0.001
*Biological processes (BP) of GO*			
Transport	15	12.500	0.014
Signal transduction	35	29.167	0.033
Cell communication	33	27.500	0.040
*Cellular components (CC) of GO*			
Transcriptional repressor complex	2	2.105	0.005
Plasma membrane	34	35.789	0.006
Actomyosin, actin part	1	1.053	0.007
Type III intermediate filament	1	1.053	0.007
Node of Ranvier	1	1.053	0.007
Symbiont-containing vacuole membrane	1	1.053	0.007
Nucleus (periNuclear)	1	1.053	0.007
Clathrin-coated vesicle	2	2.105	0.007
Activin responsive factor complex	1	1.053	0.019
Cytoplasmic vesicle	4	4.211	0.021
*Molecular function (MF) of GO*			
Adenylate cyclase activity	2	1.681	0.002
Transcription factor activity	12	10.084	0.009
Histone binding	1	0.840	0.020
Protein-tyrosine kinase activity	2	1.681	0.026

The predicted genes that overlapped in at least 7 online platforms were validated by RNA-seq data, and only 120 targets were confirmed to be inversely correlated with miR-146a-5p in expression. Enrichment analysis was conducted, adopting the 120 potential targets to identify statistically significant pathways and GO processes (p < 0.05). Most of the above terms have been reported to play essential roles in the regulation of tumorigenesis and other biological activities in cancers

**Table 5:** Predicted complementary sequence of the candidate targets and miR-146a-5p

Terms	The predicted binding sites
miR-146a-5p	3′ UUGGGUACCUUAAGUCAAGAGU
Position 1955-1961 of PRKCE 3′ UTR	5′…CUAAUGAUGACAUUCAGUUCUCU…
Position 2501-2508 of PRKCE 3′ UTR	5′…GCCUCUGGCUGGUGAAGUUCUCA…
Position 821-827 of FYN 3′ UTR	5′…AACUUACUGCGAUUUGUUCUCAA…
Position 1131-1137 of FYN 3′ UTR	5′…UUCUAUAUGUCCAGGAGUUCUCC…
Position 959-965 of AQP1 3′ UTR	5′…UCUUGCUCAUUCUUCAGUUCUCU…
Position 2566-2572 of PRKACB 3′ UTR	5′…UAUGAUCUCAGCCUCAGUUCUCU…
Position 2040-2047 of ADCY2 3′ UTR	5′…CUGGGUGGUCCACCAAGUUCUCA…
Position 666-672 of ADCY5 3′ UTR	5′…AAAUAAAACAAAACAAGUUCUCC…
Position 1994-2000 of ADCY5 3′ UTR	5′…UUUCUUCCAGCUGUUGUUCUCAA…

We identified potential binding sites of the likely targets and miR-146a-5p using TargetScan 6.1, one of the platforms available in miRWalk2.0

### AQP1 and FYN were validated by RT-qPCR to be potential targets of miR-146-5p

It has been previously proven by us that miR-146a-5p was down-regulated in lung cancer tissues [25]. Currently, we found that two likely miR-146a-5p targets, AQP1 and FYN, showed higher levels in tissues obtained from lung cancer, compared to noncancerous lung tissues (Fig. 11). The primers of AQP1 were GGACACCTCCTGGCTATTGACTAC (the forward primer) and GTTGCTGAAGTTGTGTGTGATCAC (the reverse primer); the primers of FYN were CTCAGCACTACCCCAGCTTC (the forward primer) and ATCTCCTTCCGAGCTGTTCA (the reverse primer). Hence, the expression of AQP1 and FYN tend to be inversely modulated by miR-146a-5p in lung cancer.

## Discussion

Using an integrated meta-analysis, we found that lower miR-146a-5p expression was correlated with worse outcomes in solid cancers, especially in reproductive system cancers and digestive system cancers. In particular, we observed that low expression of miR-146a-5p in gastric cancer is significantly associated with poor prognosis. Additionally, five critical pathways [PLC-mediated cascade, diacylglycerol (DAG) and inositol 1,4,5-triphosphate (IP3) signaling, AQP-mediated transport, epidermal growth factor receptor (EGFR) interaction with PLC-γ, and PLC-γ1 signaling] and several GO processes were identified as potential mechanisms underlying the effects of miR-146a-5p in cancers. Moreover, we identified six potential targets of miR-146a-5p, including *PRKCE*, *FYN*, *AQP1*, *PRKACB*, *ADCY2*, and *ADCY5*. Specifically, we have verified the expression of AQP1 and FYN in lung cancer. The high expression of AQP1 and FYN in lung cancer tissues increased the possibility of them as true targets of miR-146a-5p.

Cancer is one of the most common causes of death in humans, and rapid invasiveness and metastasis lead to an unfavorable prognosis. More than half of recent deaths in the United States are caused by lung cancer, breast cancer, prostate cancer, colorectal cancer, or pancreatic cancer in both males and females, and this can be attributed to the inability to detect tumor growth, limitations of modern imaging technologies, and insufficient bio-markers [2]. Extensive research has indicated that quite a few molecules (long non-coding RNAs, miRNAs, circRNAs, and genes) participate in the modulation of biological activities in cancers, providing a basis for improving detection and treatment [42, 43]. Therefore, we explored the clinical significance and underlying mechanisms of miR-146a-5p in diverse cancers, a miRNA with an indispensable role in the regulation of tumor progression. Our pooled results suggested that miR-146a-5p functions as a protective factor or tumor suppressor in solid cancers.

Notably, significant pooled results were found for reproductive system cancers and digestive system cancers, indicating that miR-146a-5p has prognostic value in these cancers. Several studies have demonstrated that miR-146a-5p could function as an inhibitory factor in reproductive system cancers. Sun et al. found that miR-146a-5p levels are decreased in prostate cancer, leading to tumor progression and poor prognosis; miR-146a-5p might be useful for the treatment for prostate cancer as a suppressor [20]. Additionally, miR-146a-5p has been reported to function as a powerful inhibitor in cervical cancer [44]. Similar results have been obtained for epithelial ovarian cancer [45, 46]. In this work, we found that elevated miR-146a-5p expression was indeed correlated with a better prognosis for patients with reproductive system cancers. We speculated that miR-146a-5p might be a protective factor in reproductive system cancers and is a novel therapeutic target for the improved management of tumor development. Future research is needed to evaluate this hypothesis.

Additionally, a series of studies have shown that miR-146a-5p has an indispensable role in retarding tumor progression and prolonging overall survival for patients with cancers of the digestive system. In gastric cancer, growing evidence has revealed the anti-tumor function of miR-146a-5p; it functions as a protective factor to repress neoplasm metastasis and tissue infiltration and to improve overall survival [47, 48]. Recent studies have revealed that miR-146a-5p expression is lower in pancreatic cancer tissues than in non-tumor tissues based on analyses of miR-146a-5p levels in human tissue samples, cell lines, and mouse models by real-time PCR [49, 50]. Our previous studies have confirmed that miR-146a-5p could clearly inhibit the deterioration of hepatocellular carcinoma [51, 52]. A similar protective role of miR-146a-5p has been suggested in other digestive system cancers, including colorectal cancer [53] and esophageal squamous cell carcinoma [54–56]. The results of our study were consistent with the notion that higher miR-146a-5p expression is linked to a favorable prognosis in the above cancers, especially gastric cancer. The results of our study could provide support for further research on the clinical application of miR-146a-5p in digestive system cancers.

Increased miR-146a-5p expression is beneficial for repressing the development of other cancers, especially non-small cell lung cancer (NSCLC). A recent meta-analysis concluded that the outcome of NSCLC was better in the experimental group with high levels of miR-146a-5p than in a group with low levels of expression, and indicated the potential prognostic role of miR-146a-5p [57]. Our previous work also showed that the loss of miR-146a-5p might lead to the deterioration of NSCLC with a shorter progression-free survival [25]. In head and neck squamous cell carcinoma (HNSCC) and breast cancer, miR-146a-5p was identified as a tumor suppressor [34, 58, 59]. However, we only observed a trend in which the over-expression of miR-146a-5p was related to longer survival times in NSCLC, breast cancer, and HNSCC. These associations should be explored in further studies.

We next explored the functions of predicted targets, validated predicted targets, analyzed KEGG pathways and GO enrichment, and constructed a PPI network. We found that miR-146a-5p might play a prominent role in modulating a series of biological pathways closely associated with PLC, a key enzyme on cytomembranes, including the PLC-mediated cascade, DAG and IP3 signaling, EGFR interactions with PLC-γ, and PLC-γ1 signaling. DAG and IP3 are important for the generation of phosphatidylinositol 4,5-bisphosphate and the regulation of PLC, which are linked to the release of calcium and activation of protein kinase C (PKC), two vital second messengers in signal transduction [60, 61]. Prior studies have noted the importance of PLC in modulating proliferation, invasion, and metastasis, suggesting its role in carcinogenesis and tumor progression in various malignancies [62, 63]. It has been reported that abnormal expression levels or mutations in PLC-γ (a PLC member) are related to the occurrence of breast cancer, gastric cancer, and oral squamous cell carcinoma [64–67]. We found potential interactions between EGFR and PLC-γ activities, which have a role in breast cancer, whereby EGFR/human epidermal growth factor receptor 2/PLC-γ1 signaling results in tumor cell invasion and migration [25, 68, 69]. Additionally, we found that miR-146a-5p clearly participates in the regulation of signal transduction on plasma membranes and protein-tyrosine kinase activity. We hypothesized that the specific binding sites of miR-146a-5p are located on the plasma membrane, and miR-146a-5p has a protective role, potentially by repressing the invasion and migration of tumor cells via the regulation of the above processes in solid cancers.

We found that miR-146a-5p could modulate two pathways involving AQP (regulation of water balance by renal AQPs and AQP-mediated transport). AQPs are a group of specific proteins related to the transport of water and glycerol across cell membranes; they act as promoters in cell proliferation and cell motility [70, 71]. AQPs play an indispensable role in maintaining water homeostasis in the kidney and are closely related to the regulation of urine osmolality [72]. It has been suggested that the abnormal regulation of renal AQPs would lead to diseases correlated with water balance disorders in the kidney, such as diabetes insipidus and hyponatremia [73]. Moreover, as mentioned in literature reviews, AQP expression is closely associated with tumor angiogenesis and dissemination in the majority of human malignancies [74, 75]. The application of AQP inhibitors might be beneficial to improve prognosis in diverse cancers [76]. Overall, these findings suggest that miR-146a-5p could be used to treat water balance disorders in the kidney and diverse cancers via the regulation of AQP-associated pathways.

*PRKCE* and *AQP1* were identified as two likely targets of miR-146a-5p involved in the pathways of PLC and AQPs, respectively. We also found that *FYN* is a potential hub gene in the miR-146a-5p PPI network. PRKCE, also known as PKC ε, is a novel member of the PKC isozyme family involved in the regulation of complex cellular processes in diverse cancers, as a paramount bridge between protein networks [77]. PKC ε is widely considered an oncogene, with increased expression in diverse malignant tumors, including lung cancer [78, 79], breast cancer [80], prostate cancer [81], clear cell renal carcinoma [81, 82], and HNSCC [83]. Associations have been detected between up-regulated PKC ε levels and metastatic outcomes in general cancers [84, 85]. Zhang et al. elucidated the negative modulation by miR-146a-5p f PKC ε expression, as a tumor suppressor in papillary thyroid carcinoma; PKC ε was identified as a direct target of miR-146a-5p based on a dual luciferase assay [86]. Inverse correlations between miRNAs and PKC ε were also revealed in lung cancer and HNSCC [79, 83]. Moreover, PKC ε inhibitors have been prospectively applied as novel therapies for patients with cancers [80]. In our study, *PRKCE* was identified as a potential target for miR-146a-5p; higher miR-146a-5p expression might decrease PRKCE levels to repress cellular activities in solid cancers, but further studies are needed to confirm this.

*FYN*, a member of Src family tyrosine kinases, is a proto-oncogene responsible for regulating the expression of protein-tyrosine kinases on membranes [87]. *FYN* is closely related to cancer development, tumor progression, and even dissemination in diverse cancers. Early in 2010, Yoshihito and colleagues revealed that FYN is highly expressed in prostate cancer and possibly leads to a more advanced tumor stage [88]. Elias et al. confirmed the intensive effects of FYN in promoting breast cancer development, suggesting that the over-expression of FYN is related to worse outcomes [89]. Researchers have also revealed a prospective strategy to better manage breast cancer by targeting FYN. Additionally, several studies have demonstrated that FYN functions as a promoter in diverse cancers and is associated with a poor prognosis [90–92]. In this study, we discovered higher expression levels of FYN in lung cancer than in normal tissues by conducting RT-qPCR for clinical tissues. Consequently, we speculated that miR-146a-5p might suppress the malignant transformation of cancers by controlling protein-tyrosine kinases activity, targeting FYN.

AQP1, a member of the AQP family, could modulate water transport across the plasma membrane and thereby is related to water balance in the kidney [73, 93]. Previous studies have revealed the positive regulation of AQP1 in tumor progression and demonstrated that AQP1 suppressors could repress biological activities of various tumors, including the growth of tumor cells, cell motility, and angiogenesis [94, 95]. Positive correlations between AQP1 expression and tumor development have been detected in cancers of the reproductive system (ovarian cancer [96] and prostate cancer [97]), digestive system (cancers of the stomach [98], colon [99], and esophagus [100]), and other systems (astrocytoma [101], and cancers of the lung [102], breast [103, 104], bladder [105], and pleura [106]). In our study, the high expression of AQP1 in lung cancer was proved by RT-qPCR, contrasting with low miR-146a-5p levels in lung cancer tissues in previous work [25], which strengthened the reliability of our prediction results and implied that AQP1 might be a target of miR-146a-5p in diverse cancers, especially lung cancer. Thus, it is possible that miR-146a-5p is an effective target for the management of tumor progression via AQP1, as a novel anti-cancer therapy.

As for PRKACB, ADCY2, and ADCY5. PRKACB encodes the catalytic subunit β of protein kinase A, a type of protein mainly depending on cyclic AMP (cAMP) [107]. Current studies have found the likely tumorigenic roles of PRKACB in diverse malignant tumors, including gastrointestinal cancer (gastric, colon and pancreatic tumors) and others (breast, ovary, leukemia and brain tumors) [107–109]. Moreover, PRKACB might be a promising target in cancer treatment by increasing drugs responsiveness to tumors [108, 109]. Adenylate cyclase 2 (ADCY2), a gene related to the production of cAMP, is greatly implied in acceleration of phosphor-acidification and metabolic processes of glycogen [110, 111]. Duerr et al. found that ADCY2 expression was obviously elevated in pancreatic neuroendocrine malignant tumors, possibly linked with tumor invasiveness [112]. Similar to ADCY2, ADCY5 is a catalyzer in the formation of cAMP. Takashi et al. and Chen et al. discovered that abnormal ADCY5 expression was correlated to tumor aggressiveness and DNA Methylation of ADCY5 might lead to unsatisfied outcomes for patients with lung cancer [113, 114]. We assumed that the participation of these genes in human cancers may correlate with miR-146a-5p. More biological investigation should be conducted in future to validate the correlation of three potential miR-146a-5p targets (PRKACB, ADCY2, and ADCY5) and miR-146a-5p in cancers.

However, there are a few limitations that should also be pointed out. Because hematological tumors may have different biological characteristics from solid tumors, the clinical significance and mechanism of miR-146a-5p in hematological tumors and solid tumors may be different. Because of the small number of blood tumors included in this study, the above problems could not be analyzed in detail. More samples need to be added to clarify the problem in future work. Moreover, the biological roles of miR-146a-5p in cancers and the targeting regulatory relationship between miR-146a-5p and target genes needed to be validated through further experiments in future studies. In the present study, we only chose AQP1 and FYN for qRT-PCR validations. RT-qPCR experiments for all potential targets in various cancer types should be performed in future studies to achieve a comprehensive verification.

## Conclusion

Based on a comprehensive meta-analysis and bioinformatics analysis, we concluded that miR-146a-5p might serve as an inhibitory factor in general cancers (reproductive system cancers and digestive system cancers, especially gastric cancer). We speculated that miR-146a-5p might inhibit the progression of solid cancers via pathways involving PLC (candidate targets: PRKCE and FYN) or AQPs (candidate target: AQP1) by inversely regulating target genes. Moreover, miR-146a-5p could be used to treat certain diseases correlated with water balance disorders in the kidney by regulating kidney AQP pathways. In addition, miR-146a-5p could repress certain biological activities in tumor cells by the modulation of cell communication on plasma membranes. Overall, miR-146a-5p could be utilized as a prognostic biomarker, with implications for the prediction and treatment of diverse cancers.

## Data Availability

The datasets used and/or analysed during the current study are available from the corresponding author on reasonable request.

## Abbreviations

TCGA: The Cancer Genome Atlas
GEO: gene expression omnibus
HRs: extract hazard ratios
95% CIs: 95% confidence intervals
DE genes: differentially expressed genes
GO: gene ontology
STRING: search tool for the retrieval of interacting genes/proteins
PPI: protein–protein interaction
KEGG: Kyoto Encyclopedia of Genes and Genomes
PLC: pathways involving phospholipase C
AQPs: aquaporins
DAG: diacylglycerol
EGFR: epidermal growth factor receptor
PKC: protein kinase C
HNSCC: head and neck squamous cell carcinoma
GC: gastric cancer
TNBC: triple negative breast cancer
NSCLC: non-small cell lung cancer
DLBCL: diffuse large B cell lymphoma
NK/T: NK/T cell lymphoma
ALL: acute lymphoblastic leukemia
AML: acute myeloid leukemia
qRT-PCR: quantitative real-time polymerase chain reaction
MISH: miRNA in situ hybridization
BLCA: urothelial bladder cancer
BRCA: breast invasive cancer
CESC: cervical cancer
COAD: colon adenocarcinoma
ESCA: esophageal cancer
GBM: glioblastoma multiforme
HNSC: head and neck squamous cell carcinoma
KIRC: kidney renal clear cell carcinoma
KIRP: kidney renal papillary cell carcinoma
LAML: acute myeloid leukemia
LGG: lower grade glioma
LIHC: liver hepatocellular carcinoma
LUAD: lung adenocarcinoma
LUSC: lung squamous cell carcinoma
OV: ovarian serous cystadenocarcinoma
PAAD: pancreatic adenocarcinoma
READ: rectum adenocarcinoma
SARC: sarcoma
SKCM: skin cutaneous mMelanoma
STAD: stomach adenocarcinoma
UCEC: uterine corpus endometrial carcinoma
